# Liposomes-in-Hydrogel Delivery System with Mupirocin: *In Vitro* Antibiofilm Studies and *In Vivo* Evaluation in Mice Burn Model

**DOI:** 10.1155/2013/498485

**Published:** 2013-11-28

**Authors:** Julia Hurler, Karen K. Sørensen, Adyary Fallarero, Pia Vuorela, Nataša Škalko-Basnet

**Affiliations:** ^1^Drug Transport and Delivery Research Group, Department of Pharmacy, University of Tromsø, Universitetsveien 57, 9037 Tromsø, Norway; ^2^Vascular Biology Research Group, Department of Medical Biology, University of Tromsø, Universitetsveien 57, 9037 Tromsø, Norway; ^3^Pharmaceutical Sciences, Department of Biosciences, Abo Akademi University, Artillerigatan 6A, 20520 Åbo, Finland; ^4^Division of Pharmaceutical Biology, Faculty of Pharmacy, University of Helsinki, P.O. Box 56, 00014, Finland

## Abstract

Previously, we have proposed mupirocin-in-liposomes-in-hydrogel delivery system as advanced delivery system with the potential in treatment of burns. In the current studies, we evaluated the system for its cytotoxicity, ability to prevent biofilm formation, act on the mature biofilms, and finally determined its potential as wound treatment in *in vivo* mice burn model. The system was found to be nontoxic against HaCaT cells, that is, keratinocytes. It was safe for use and exhibited antibiofilm activity against *S. aureus* biofilms, although the activity was more significant against planktonic bacteria and prior to biofilm formation than against mature biofilms as shown in the resazurin and the crystal violet assays. An *in vivo* mice burn model was used to evaluate the biological potential of the system and the healing of burns observed over 28 days. The *in vivo* data suggest that the delivery system enhances wound healing and is equally potent as the marketed product of mupirocin. Histological examination showed no difference in the quality of the healed scar tissue, whereas the healing time for the new delivery system was shorter as compared to the marketed product. Further animal studies and development of more sophisticated *in vivo* model are needed for complete evaluation.

## 1. Introduction

Burn wound healing is a complex process characterized by challenges in wound management, treatment, and healing in terms of scarring [1]. Although the improvements in burn care increased the survival of burned patients, they also resulted in prolonged hospital stays [2]. One of the recent focuses of improved wound therapy, including burn therapy, is the use of antimicrobials, not only to prevent the wound infection, but also to act on biofilms [3, 4]. At the same time, topical antimicrobial formulations need to be nontoxic and nonirritant to the skin.

In previous studies we developed and characterized the mupirocin-in-liposomes-in-hydrogel system destined for burns therapy [5]. Chitosan, which is the basic vehicle in our delivery system, is known to exhibit very low toxicity potential in humans. Mupirocin is an effective and safe antimicrobial used in topical wound treatment [6]. The safety of mupirocin-in-liposomes destined for skin application was tested in this study.

Many bacteria that are known to colonize wounds, including burns, are also known to be able to form biofilms, such as *Staphylococcus aureus* and *Pseudomonas aeruginosa* [7]. Biofilms are 3D-aggregates characterized by an increased resistance to antimicrobial, immunological, and chemical attack, making their eradication a challenge [8]. Wounds are typically subject to exogenous and endogenous microbial contamination, particularly pronounced by the presence of maturing bacterial biofilms in the wound. A typical challenge of these infections is the presence of the extracellular polymeric material (commonly referred to as matrix or EPS) that encases the cells, creating a physical barrier that makes access to the cellular core more difficult and contributing to the overall stability and persistence of the biofilms. The presence of wound biofilm may further inhibit downregulation of the immune responses causing systemic debilitation [9–11].

Topical antimicrobials are an appealing choice for the treatment of burn wound infections, due to a decreased risk of systemic toxicities and the ability to deliver the antimicrobials directly to the site of action. They are employed for both prophylaxis and treatment of burn wound infections despite no established susceptibility breakpoints. Mupirocin is known to be effective against suspended gram positive bacteria, including MRSA [2]. Additionally, antibiofilm effects of mupirocin against *S. aureus *have been reported [12]. The aim of this work was to examine the effect of mupirocin, formulated in liposomes, against *S. aureus *biofilms. We hypothesized that liposomes could enhance the antibiofilm activity of mupirocin by increasing its penetrability into biofilms through the EPS and probably even deeper into the biofilms, while at the same time reducing its effects on the planktonic bacteria, thus potentiating the antibiofilm selectivity of mupirocin. Antibiofilm drugs with lower effects on planktonic bacteria are particularly promising, as they can potentially lower the likelihood of bacterial resistance development.

The final efficacy evaluation of any wound dressing needs to be performed in an appropriate animal model. As our focus was the potential of mupirocin in wound treatment, we selected the mice burn model and compared the efficacy of our system against marketed mupirocin-containing product, Bactroban, a commercially available product used for topical treatment of burn wounds. According to producer recommendations, Bactroban is to be applied on the wound three times a day. Our delivery system has shown to provide sustained release [5] and can thus lessen the number of applications per day, which results in reduced nursing time and less discomfort to the patient due to the dressing changes [13]. The tested delivery system is based on chitosan hydrogel as vehicle and our aim was, in addition, to confirm that chitosan itself has the wound healing potential, which would act in synergy with antimicrobial and liposomes in the system.

## 2. Materials and Methods

Mupirocin calcium dehydrate micronized (MC) was kindly provided by Pliva (Zagreb, Croatia). High MW chitosan (Brookfield viscosity 800.00 cps and degree of deacetylation of 77%) was a product of Sigma Aldrich Chemistry (St. Louis, MO, USA). Lipoid S 100 was a gift from Lipoid GmbH (Ludwigshafen, Germany). Glycerol and acetic acid were obtained from Merck KGaA (Darmstadt, Germany). Propylene glycol was purchased from the Norwegian Medical Depot (Oslo, Norway). Bactroban creme (2%, w/w) was the product of GlaxoSmithKline (Barnard Castle, Great Britain).

### 2.1. Preparation and Characterization of Liposomes

Liposomes were prepared according to the dry film method as described previously [5]. In a round bottom flask, mupirocin (30 mg) and phospholipid (200 mg) were dissolved in methanol (~20 mL). The solvent was then removed by vacuum on a rotary evaporator (Büchi R-124, Büchi Labortechnik, Flawil, Switzerland). The drug-lipid film was rehydrated by 10 mL of distilled water (pH 6.8) and hand shaken for 10 min. The liposome dispersion was left in the refrigerator overnight before removal of unentrapped mupirocin by ultracentrifugation (25 min at 10°C and 32000 rpm Beckmann-L8-70M ultracentrifuge, Beckmann instruments Inc., Palo Alto, CA, USA). The pellet was then resuspended in 10 mL of distilled water; liposomes that were destined for hydrogel formulations were resuspended in 5 mL of distilled water.

For calculating the entrapment efficiency of mupirocin in liposomes, mupirocin concentration in both pellet and supernatant was measured by high performance liquid chromatography according to the method described in Hurler et al. [5]. In brief, a reversed phase column (XTerra RP_18_ 5 *μ*m, 3.9 × 150 mm^2^ column, Waters, Dublin, Ireland) was used in combination with a mobile phase that consisted of acetonitrile and ammonium acetate (0.05 M) in the ratio of 27.4 : 72.5 (v/v), and pH value was adjusted to 6.3 with hydrochloric acid. The flow rate was set to 1 mL/min, column temperature was 30°C, and sample temperature was 25°C. The samples (injection volume of 20 *μ*L) were run for 9 min, at detection wavelength of 228 nm.

The liposomal suspensions showed an entrapment efficiency of 62.4% (±8.8%) and a mupirocin concentration in liposomal suspension of 1.87 mg/mL. Liposomal suspensions for hydrogel preparations had accordingly a mupirocin concentration of 3.74 mg/mL, as resuspension medium was reduced to half. Liposomes were multilamellar in structure and rather polydisperse (PI of 0.570), with an average diameter of 920 nm, as determined by photon correlation spectroscopy [5].

### 2.2. Preparation of Chitosan Hydrogel and Liposomes-in-Hydrogel

Chitosan hydrogel was prepared as described earlier [15]. In brief, high molecular weight chitosan (2.5%, w/w) was dissolved in a blend of glycerol (10%, w/w) and acidic acid (2.5%, w/w), respectively. The gel was left at room temperature for 48 h before further treatment.

For liposomes-in-hydrogel formulations, 10% (w/w) of liposomal dispersion was manually mixed into chitosan hydrogel until even distribution. The resulting mupirocin concentration was at 374 *μ*g/mL formulation.

### 2.3. Cell Viability Testing

Cell viability was tested according to the modified method by Kempf et al. [16] and Louis and Siegel [17]. HaCaT cells were seeded in 24-well plates and solutions of free mupirocin and mupirocin-in-liposomes (1, 5, 10, 50, and 100 *μ*g/mL, resp.) were added. DMSO, which was used as solvent for mupirocin and empty liposomes served as controls. Plain medium served as a negative control. After 24 h of incubation at 37°C and 5% CO_2_, the cells were washed with RPMI medium to remove the dead cells. The remaining cells were trypsinated and, after 2 min at 37°C, washed again with medium. The cell suspension with trypan blue was incubated for 2 min at 37°C and afterwards counted in a haemocytometer. The dead cells appeared blue due to dye, whereas the viable cells remained unstained. The cell viability was calculated by subtracting the amount of dead cells from the total number of cells and was expressed as percentage. The results were set into a relation with the negative control. All tests were performed in triplicate.

### 2.4. Biofilms

#### 2.4.1. Bacterial Growth in Planktonic and Biofilms

The biofilm-producing strain *Staphylococcus aureus *(ATCC 25923) was used as a model bacterium. Bacteria were cultured in tryptic soy broth (TSB) (Fluka Biochemika, Switzerland) under aerobic conditions at 37°C and 200 rpm to reach exponential growth (10^8^ CFU/mL). The planktonic trials were performed by adding exponentially grown bacteria (10^6^ CFU/mL) into sterile, flat-bottomed, 96-polystyrene microtiter well plates (Nunclon Δ surface; Nunc, Denmark) together with the samples and incubated during 18 h at 37°C, similar time as the one used for the biofilm formation. The absorbance was automatically measured at 620 nm every 15 min using a Varioskan multimode plate reader (with the aid of a kinetic loop) and an automatic plate shaking step (240 rpm, 5 s) was performed prior to each absorbance measurements. Endpoint measurements obtained after 18 hours were used for the potency calculations. To promote biofilm formation, the exponentially grown bacterial suspension (10^6^ CFU/mL, 200 *μ*L/well) was added into sterile 96-polystyrene microtiter well plates and the plates were incubated for 18 h under aerobic conditions (37°C, 200 rpm). During the preventive antibiofilm screenings, compounds were added at the same time as the bacterial suspension into the wells. To measure the effects on the mature biofilms, they were allowed to form first for 18 h, and then the planktonic solution in each well was replaced with fresh TSB containing the compound, followed by incubation for additional 24 h (37°C, 200 rpm).

#### 2.4.2. Sample Preparation

The following samples were tested: (i) empty liposomes (20 mg/mL lipid content in distilled water, coded as EL) used as controls, (ii) liposomes loaded with mupirocin (20 mg/mL lipid, 1.729 mg/mL mupirocin calcium dehydrate, in distilled water, coded as LM), and (iii) free mupirocin in propylene glycol (1.729 mg/mL mupirocin calcium dehydrate, in propylene glycol 10% (w/w), coded as M). The samples were let to stand at room temperature for 30 min before starting the trials. To ensure complete homogenization, the samples were sonicated in a high power ultrasonic bath (Bandelin Sonorex Digitec) at RT (5 min, 35 kHz). Compound concentrations were within the range 0.01–70 *μ*M for the prior-to-exposure trials and within the range 1–405 *μ*M for the postexposure trials. Penicillin G was used as a positive control in both the biofilms and planktonic trials. A stock solution of penicillin G (20 mM) was freshly prepared before the trials in a Mueller-Hinton media.

#### 2.4.3. Biofilm Quantification Assays

For measuring the presence of viable cells and the total biomass in the wells, the resazurin staining and crystal violet were used, respectively, using the protocols that have been described earlier [18, 19]. Briefly, the biofilms were first stained with 20 *μ*M resazurin for 20 min (200 rpm, in room temperature, RT), and fluorescence was measured at *λ*_excitation_ = 560 nm and *λ*_emission_ = 590 nm using a Varioskan multimode plate reader. Immediately after, the resazurin stain was removed and replaced by crystal violet. The biofilms were stained for 5 min (RT) using a Combi multidrop dispenser then washed two times with milli Q-water using a Biomek 3000 liquid handling workstation. The remaining dye was then solubilized in 96% ethanol and absorbance (*λ* = 595 nm) was measured using a Varioskan multimode plate reader.

#### 2.4.4. Data Processing

Potencies (as expressed through half-inhibitory concentrations, IC_50_ values) were determined by a nonlinear regression curve fitting (with variable slope) using GraphPad software, Prism 5.0c for Mac OS X, USA (2011). At least 8 concentrations were included in the potency trials, and each concentration was tested with 8 replicates in 3 independent trials. The performance of the antibiofilm assays was evaluated using typical statistical parameters (*Z*′, *S*/*B* and *S*/*N*). Their calculations were made with the aid of the control samples (untreated biofilms and media control samples). The parameters were calculated as in [18].

### 2.5. * In Vivo* Experiment

#### 2.5.1. Animals

The aim of *in vivo* study was to evaluate the effectiveness of the mupirocin-in-liposomes-in-hydrogel system as wound dressing as well as compare its effectiveness for a marketed product. Prior to the experiment, 7-week-old CD1 male mice (*n* = 35) were purchased from Charles River Laboratories, Sulzberg, Germany. The animals were randomly divided into five groups each receiving different treatment: (I) Bactroban 2%, (II) liposomal mupirocin, (III) chitosan hydrogel, (IV) mupirocin-in-liposomes-in-hydrogel, and (V) no treatment, respectively. The mice were kept in individual filter-top cages and were allowed feed and water *ad libitum* during the one week of acclimatization and throughout the experiment. The study was approved by the Norwegian National Animal Research Committee; all experiments were performed according to the recommendations of the Federation of European Laboratory Animal Science Associations and the Norwegian legislation on care and use of experimental animals. The mice were housed in the same room at 21°C and 55% relative humidity under a 12 h day/-night cycle.

#### 2.5.2. Burn Wounds

Each mouse was weighed for the health monitoring reasons before it was anaesthetized by 2% isoflurane gas (mask) and received a subcutaneous injection of Temgesic (0.3 mg/kg) for analgesia. The animal was shaved at its dorsal side with an electric razor and disinfected using 70% ethanol (v/v). The burn wound was induced by pressing a cylindrical metal rod (0.79 cm^2^) that has been heated on a warming plate for 30 s, to the shaved area for 30 s. This procedure led to a burn wound of approximately 2% of body surface. The wound was photographed for the measurement of wound size reduction.

Animals received subcutaneous injected Temgesic (0.3 mg/kg) every 12 h for the first 48 h of the experiment; afterwards all mice received Metacam (5 mg/kg) *per os* in their drinking water for the additional four days.

#### 2.5.3. Treatment of Wounds

As the treatment, 1 g of different formulations was applied directly onto the burn wound. Group I animals were treated with Bactroban 2% (*n* = 4); Group II was treated with liposomal mupirocin (*n* = 5); Group III with plain chitosan hydrogel (*n* = 4); Group IV with mupirocin-in-liposome-in hydrogel (*n* = 6), and Group V served as control group (*n* = 4) and did not receive any treatment.

The first treatment was applied right after induction of the burn wound. During the first week after burn induction, mice received treatment once daily; in the remaining three weeks the treatment was applied every second day.

#### 2.5.4. Evaluation of Burn Wound

The size of the wound was measured at days 4, 8, 12, 16, 20, 24, and 28 after the burn induction.

On day 28, the animals were sacrificed and histological samples were taken from the healed wound site and from the normal skin (dorsum) and fixed in 4% paraformaldehyde in PBS with 0.02 M sucrose, pH 7.4. The tissue samples were embedded in paraffin wax, processed, sectioned (3–5 *μ*m), and stained with hematoxylin and eosin (H&E), or with the van Gieson technique for connective tissue staining.

#### 2.5.5. Analysis of Data

All data are presented as the mean ± standard deviation (SD). Data were analyzed by analysis of variance repeated measures (rANOVA); a *P* value < 0.05 was regarded as significantly different. Tukey's multiple-comparison post hoc test was applied when applicable.

## 3. Results and Discussion

### 3.1. Cell Viability

Formulations that are destined for administration onto burn wounds have to fulfill several requirements. The primary one is that these formulations should not be toxic to healthy skin cells. In this study we tested the cell viability of HaCaT cells, that is, immortalized keratinocytes, when exposed to either free mupirocin or mupirocin-in-liposomes. Whereas it is know that mupirocin is nontoxic in humans [6], liposomal mupirocin formulations have not been, to the best of our knowledge, tested regarding their toxicity on the skin.

After exposure for 24 h [16], mupirocin both in the free form and encapsulated in liposomes was found to be noncytotoxic (0–10%) or moderately cytotoxic (>10–20%) in the tested concentration range from 1 up to 100 *μ*g/mL. Neither DSMO, even in the highest concentration used, nor empty liposomes exhibited any cytotoxic effect; the survival rate of cells after 24 h was 95%. Free mupirocin did not exhibit any cytotoxic effect in any of the tested concentrations. Mupirocin-in-liposomes showed a similar pattern with the exceptions at the concentrations of 5 and 50 *μ*g/mL. However, as the cell survival at the highest tested concentration (100 *μ*g/mL) was over 96%, the results observed that for those two concentrations 5 and 50 *μ*g/mL (cell survival 81.5 and 86.6%, resp.) can only be explained by the failure in experimental setup.

In this study we also showed that mupirocin-in-liposomes exhibited no toxic effect on keratinocytes. Boyce et al. [20] could not show significant growth inhibition of keratinocytes and fibroblasts when exposing the cells to a blending of neomycin (40 *μ*g/mL), polymyxin B (700 U/mL), and mupirocin (40 *μ*g/mL). Other agents that are widely used in wound dressings, such as silver, can exhibit significant toxic effect on both keratinocytes and fibroblasts [21]. Burd et al. [21] tested various wound dressings containing silver for their cell toxicity and reepithelialization and found that dressings such as Acticoat and Contreet Foam exhibit significant toxic effects on keratinocytes and fibroblasts that were isolated from human skin. When tested in *in vivo* experiments these dressings were reported to delay the reepithelialization. However, cytotoxicity tests of Kempf and coauthors [16], who used HaCaT cells instead of keratinocytes cultured from excised human skin that Burd and colleagues [21] were using, could not confirm the cytotoxic effect of Acticoat to the same extent. These inconsistent findings indicate that the cell type is influencing the cell viability properties.

### 3.2. Biofilm

The complexity of bacterial communities in wounds has historically been underestimated and only recently the presence and importance of biofilms in wound healing has started to gain a wider recognition [22]. A biofilm is a complex microbial community, built by the bacteria embedded in a protective matrix of sugars and proteins. Biofilms contribute to the persistent, chronic inflammatory changes in the wound bed and are clearly linked to impaired wound healing [9–11]. Although *in vitro* and *in vivo* models of biofilms vary and remain to be subject for discussions, the models intent to simulate the functional characteristics of chronic pathogenic biofilms and can be sampled for characterization and analysis of the experimental biofilms [8]. However, the current lack of adequate *in vivo* models and their proper validation, limits the possibility of mimicking the wound, particularly burn, completely [22].

Mupirocin is known to be effective against suspended gram positive bacteria, including MRSA. In our previous study on antimicrobial activity of various mupirocin formulations, such as free mupirocin, mupirocin-in-liposomes, mupirocin-in-liposomes-in-hydrogel, and a marketed product (Bactroban), we confirmed their activities against *S. aureus *[5]. It is known that many bacteria are able to form biofilms, including *S. aureus*, which is amongst the typical bacterial species colonizing burn wounds [7, 23].

**Figure 1:**
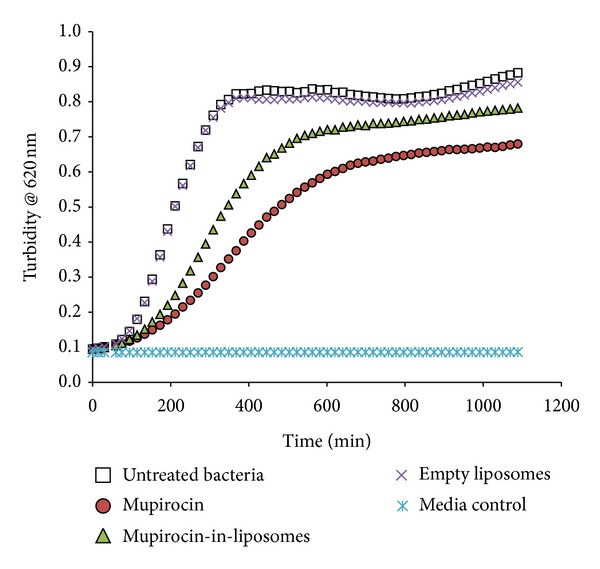
Kinetic curves of the effects of mupirocin samples on planktonic growing of *S. aureus*. The effects of the mupirocin samples are shown only at 0.1 *μ*M for simplicity.

In this study the potential of mupirocin, both in the free form and in liposomes, was tested for its ability to prevent or counteract established *S. aureus* biofilms. Firstly, we showed that free mupirocin (M) is indeed active against planktonic *S. aureus *with potency values on the submicromolar range (Table 1) and thus has a potential to prevent the creation of biofilms. The mupirocin-in-liposomes (LM), in contrast, was found to be 2.7 times less active, based on endpoint measurements made after 18 hours of bacterial growing. In addition, the LM sample was less effective in delaying the planktonic growing, as seen on the kinetic curves of planktonic growth (Figure 1). These results support the view that the liposome formulation was less available to interact with the bacteria, resulting in a lower activity of the antibiotic on the suspended bacteria. Empty liposomes, as expected, did not have any effect on the planktonic growth.

The antibiofilm effects of the mupirocin samples were then measured (Table 1). These results refer to the effects on biofilms viability as measured using the resazurin reduction assay. In brief, this assay is based on the use of a redox dye (resazurin) which is converted to a fluorescent product, resorufin, by the metabolically active bacteria residing on the inner core of the biofilms. Both samples (M and LM) were able to prevent biofilm formation, and the potency values in both cases corresponded rather well with the potency determined in planktonic bacteria. In this exposure paradigm, it is plausible to hypothesize that both M and LM interact with planktonic bacteria and efficiently decrease the amount of viable bacteria that can reach the surface and initiate the biofilm formation process. Similar activity trends were observed in the crystal violet assay, which indicated that both M and LM had concomitant effects on the viability and biomass of the biofilms. The biomass reduction is, in this case, probably related to the decrease on the viable cell fraction of the biofilms.

Measurements of mupirocin effects on the established (18 h-formed) biofilms were then performed, but the registered antibiofilm activity did not exceed 50% for both M and LM samples, thus hampering the estimations of their potencies (as expressed by IC_50_ values). For instance, in the case of LM at 1 *μ*M concentration, a 40% inhibition of biofilm viability in mature biofilms was seen, but the inhibition did not increase over 50% even with as high concentrations as 405 *μ*M. This situation is similar to what has been previously registered on biofilms exposed to penicillin (Table 1). In the case of penicillin-treated biofilms, we have earlier demonstrated that a significant overproduction of the matrix takes place, which could impede the access of penicillin to the remaining metabolically active cells [14]. Alternatively, it could be possible that in penicillin or mupirocin-insulted biofilms a higher fraction of the cells could switch to a dormant or less metabolically active state, in which they are less sensitive to the effects of these antibiotics. In such scenario, increasing the penetrability of the mupirocin by means of the liposomes carriers would not influence the antibiofilm activity, as we had initially hypothesized.

Another study has shown the reduction of *S. aureus *(ATCC 25293) biofilm biomass with mupirocin in concentration between 15 and 250 *μ*M by 90% [12]. The discrepancy between these results may be explained with the different methodology that was used. In our study the mature biofilms were allowed to grow for 18 h before the sample was added, Ha et al. [12] let the biofilm grow for 8 days. It is known that time also influences the susceptibility of biofilms against antimicrobials and young biofilms (as the ones formed in our *in vitro* model) may respond differently to the effects of antibiotics when compared to older biofilms. In an *in vivo* study, Roche et al. [24] have shown that mupirocin was effective in partial-thickness wounds that were infected with methicillin-resistant *S. aureus* (ATCC 33592). However it was more effective when applied 4 h after the bacterial infection as compared to the application 24 h after bacterial infection. These findings support the results in our study and show the importance of early treatment in order to avoid the formation of biofilms on wounds. In addition, other studies confirmed the antibiofilm effectiveness of mupirocin not only against *S. aureus*, but also against *P. aeruginosa* [25] which is a bacterial specie also typically found in wounds, colonizing the deeper areas of the affected issue.

### 3.3. *In Vivo* Evaluation

**Figure 2:**
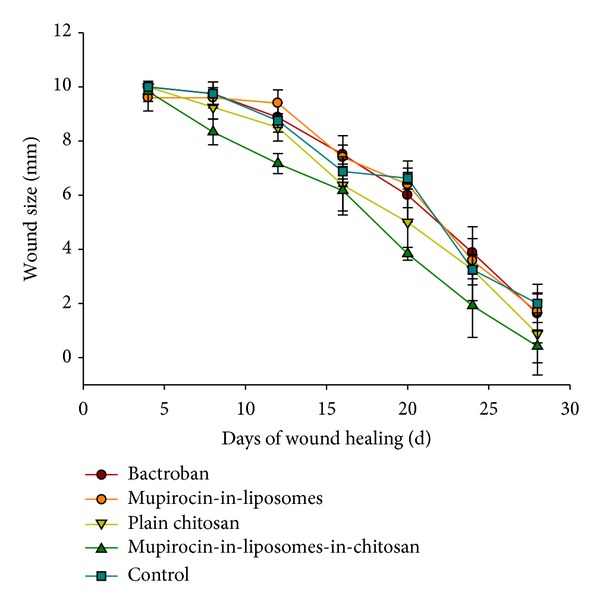
Wound size reduction in *in vivo* healing experiments.

**Figure 3:**
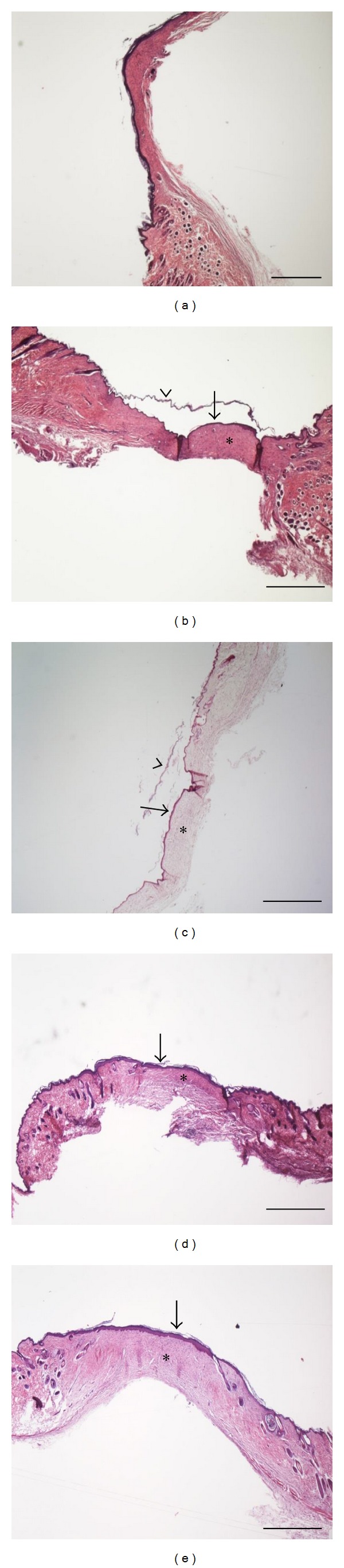
Histological photographs of burn wounds, 28 days after induction. Sections are stained with hematoxylin and eosin. Scale bars: 1 mm. Representative samples are shown from animals treated with (a) Bactroban, Group I; (b) mupirocin-in-liposomes, Group II; (c) plain chitosan hydrogel, Group III; and (d) mupirocin-in-liposomes-in-hydrogel, Group IV. (e) shows a healed burn wound from a nontreated mouse (Group V, control). Arrows point to epidermis, and (∗) indicates dermis in (a)–(e). Arrow heads in (b) and (c) point to the keratin layer which often detached during tissue preparation.

In this study we evaluated different mupirocin formulations in a mice burn wound model; for this purpose burn wounds (1 cm in diameter) were induced on the dorsal side of mice and treated for four weeks. The change in wound size over time with according formulations is displayed in Figure 2 (see also Supplementary Materials available online at http://dx.doi.org/10.1155/2013/498485). On day 28 after the induction of burn wounds the mice were sacrificed and the scar tissue was evaluated histologically (Figure 3).

All wounds were closed at latest after 28 days of treatment; with mupirocin-in-liposomes-in-chitosan showing significantly (*P* < 0.05, according to Tukey's multiple-comparison test) faster healing compared to the other groups. However, one needs to consider that all groups, the group I, which was treated with Bactroban, the group II (mupirocin-in-liposomes), and the group III (plain chitosan hydrogel) had similar healing time as compared to the control group V, which did not receive any treatment. This would implement that the healing time of the wounds alone cannot be the dominant indicator when evaluating the quality of burn treatment. We did not observe any signs of inflammation during the tested period, which was also confirmed in histological evaluation. We need to state that one of the reasons that we failed to show the significance among the different treatment efficiencies can be the reduced number of animals completing the experiments (66%), as animals biting of the wounded site were euthanized and excluded from the experiment. Male mice tend to be more aggressive than female mice, and it may therefore be a better option to use female mice.

The histological evaluations of the quality of the scar tissue showed complete re-epithelialization of all wounds. In all groups, the epidermis of the healed wounds was typically thin, hairless, and without epidermal ridges, while the underlying dermis contained a dense connective tissue devoid of inflammation (Figure 3). In group III (plain chitosan hydrogel, *n* = 4), two animals showed increased epidermal thickness and ridge formation in part of the wound, covering areas in dermis with chronic inflammation and calcification, which might be a result of itching and scratching by the animals. One animal in group I (Bactroban, *n* = 4) showed a complete recovery of hair follicles in the healed wound (not shown). We have also realized that the final histological evaluation was not sufficient to conduct complete comparison and are planning to design further studies in a different time frame, namely, performing the histological evaluation at earlier time points, every seven days.

Finally, the burn model we have used has several limitations [26] and needs to be optimized prior to further studies. The challenge of developing a suitable and reproducible animal model for burns remains to be addressed [3] but these methodological elements that we have discussed above could be taken into account to help improving future *in vivo* studies in this burn wounds model.

## 4. Conclusions

We have shown that the novel drug delivery system for mupirocin, mupirocin-in-liposomes-in-hydrogel, is nontoxic in skin cells, at least in the tested HaCaT cells. It exhibits the potential to prevent the formation of *S. aureus*-based biofilms which is of high relevance concerning the development of wound dressings. Although we could not prove the superiority of this system over the marketed product in *in vivo* mice burn model, we have shown that the system is equally good and safe for administration onto wounded site. More *in vivo* evaluations are needed to define the systems' potential as wound dressing.

## Supplementary Material

Burn wounds at different time points of healing.

## Figures and Tables

**Table 1:** Quantification of antibacterial and antibiofilm activity of mupirocin samples.

Sample	Biofilm bacteria IC_50_, *μ*M (95% confidence intervals)		Planktonic bacteria IC_50_, *μ*M (95% confidence intervals)
	*Prior to* biofilm formation	*After* biofilm formation	Turbidity
	Viability	Viability	
Mupirocin	0.27 (0.48–0.70)	50%^a^	0.20 (0.42–0.67)
Mupirocin-in-liposomes	0.58 (0.16–0.45)	50%^a^	0.53 (0.14–0.26)
Penicillin G	0.13 (0.12–0.14)	45.2%^b^	0.10 (0.08–0.13)

^a^Percentual inhibition at 405 *μ*M.

^b^Percentual inhibition at 5 mM. Penicillin G fails to cause more than 45.2% of inhibition of biofilms viability in the postexposure assay, as previously shown in Skogman et al. [14].
