# Supplementary figures and images for: Liposomes-in-Hydrogel Delivery System with Mupirocin: In Vitro Antibiofilm Studies and In Vivo Evaluation in Mice Burn Model
Source: Biomed Res Int. 2013 Nov 28;2013:498485. doi: 10.1155/2013/498485 (PMC3863504; doi:10.1155/2013/498485)

**Supplementary material:**


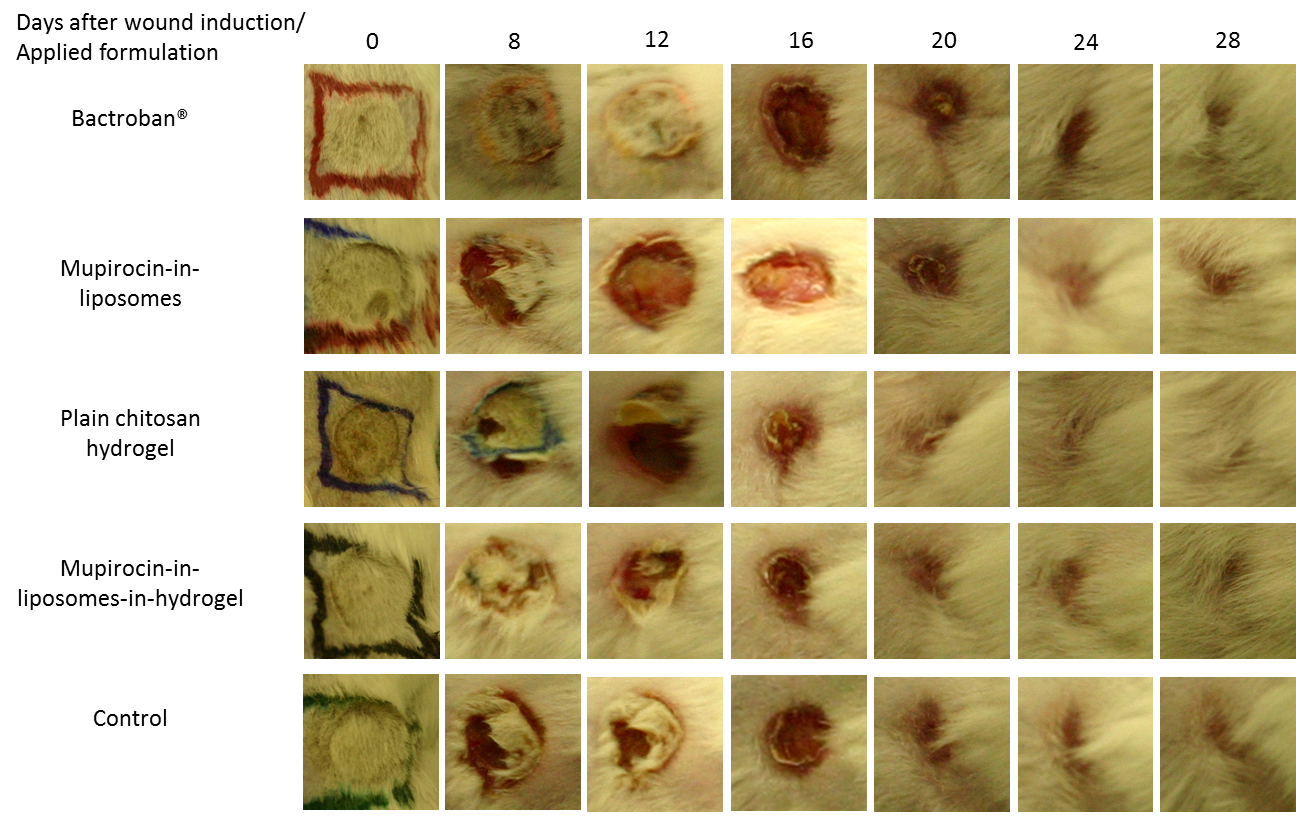


Burn wounds at different time points.

Supplement: Supplementary file 1 — Burn wounds at different time points of healing. [file 498485.f1.docx]
